# *FOXP2* gene and language development: the molecular substrate of the gestural-origin theory of speech?

**DOI:** 10.3389/fnbeh.2013.00099

**Published:** 2013-08-05

**Authors:** Carmelo M. Vicario

**Affiliations:** School of Psychology, The University of QueenslandBrisbane, QLD, Australia

The view that language evolved from a primarily gestural mode of communication has its roots on the 18th-century philosophers speculations (Vico, 1953/1744; de Condillac, 1971/1756).

Over time, these philosophical thoughts have gradually got consistence, thanks to the research in the field of Psychology and Neuroscience, which has provided exciting evidence in support of the so-called gestural origin-theory of language (Corballis, 2002). This theory recognizes to the gestures a precise role in language development. In particular, in terms of evolution it has been suggested that spoken language evolves from an ancient communication system using arm gestures. Accordingly, it has been suggested that gestures of the mouth might have been added to the manual system to form a combined manuofacial gestural system (Corballis, 2002; Gentilucci and Corbalis, 2006).

The literature on Mirror Neurons has provided a strong support to the gestural-origin theory thanks to the evidence of a close relationship between arm (Gallese et al., 1996; Rizzolatti et al., 1996) and mouth actions (Ferrari et al., 2003) in the brain of non-human primates. In particular, it has been shown that the area F5 of the monkey premotor cortex includes also a class of neurons that discharge when the animal grasps an object with the either the hand or the mouth (Rizzolatti et al., 1988). However, although a recent meta-analysis of 125 human fMRI studies (Molenberghs et al., 2012) identified a core network of human brain regions that possess mirror properties associated with action observation and execution, there is also a literature that challenges the existence of these neurons in humans. In fact, direct evidence for the existence of mirror neurons in humans is still lacking (Dinstein et al., 2008; Hickok, 2009). On the other hand, some studies provide data against it. For example, the study of Lingnau et al. (2009) failed in finding adaptation for motor acts that were first executed and then observed (as found for executed motor acts, when these were preceded by execution or observation of the same motor act) in the brain areas that are typically considered as endowed of mirror properties. This implies that the link between motor gestures and spoken language is not necessarily mediated by neurons provided of “mirror” properties.

Clues in support of the gestural-origins theory of the language are also provided by the research on humans. For example, grasping larger objects (Gentilucci et al., 2001) and bringing them to the mouth (Gentilucci et al., 2004) induces selective increases in parameters of lip kinematics and voice spectra of syllables pronounced simultaneously with action execution. Moreover, it has been reported that repetitive transcranial magnetic stimulation of Broca's area affects verbal responses to gesture observation (Gentilucci et al., 2006). This suggests that Broca's area is probably involved in the simultaneous control of gestures and word pronunciation.

Neuroimaging studies on humans provide a further support to this direct link between gestures and verbal language. Sakai et al. (2005) used functional Magnetic Resonance Imaging to examine hemispheric dominance during the processing of signed and spoken sentences. Their study was provided of two conditions: (i) the sign condition with sentence stimuli in Japanese Sign Language (JSL) in which were tested Deaf signers of JSL, hearing bilinguals (children of Deaf adults, CODA) of JSL and Japanese (JPN); (ii) the speech condition in which were tested hearing monolinguals (Mono) of JPN with auditory JPN stimuli alone (AUD), or with an audiovisual presentation of JPN and JSL stimuli (A and V). The authors found that the ventral part of the left inferior frontal gyrus (F3t/F3O) showed no main effects of modality condition, providing evidence in support of the existence on a common area for the processing of linguistic information from both signed and spoken sentences. Moreover, it has been recently documented the common involvement of the left area 7A in the superior parietal lobule while performing a sequenced button presses task or a sequence of different syllables repetition task (Heim et al., 2012). These data demonstrate the existence of a common cortical module in the area 7A while sequencing vocal gestures and hand motor actions.

Finally, a support to the gestural-origin theory of language originates from the study of human infants. For example, Fogel and Hannan (1985) provided evidence of gesture-vocalization synchrony in 2-and 3-months-old human infants. Word comprehension in children between 8 and 10 months and word productions between 11 and 13 months are typically accompanied by deictic gestures (Volterra et al., 1979; Bates and Snyder, 1987). Deictic gestures (referring to an object or location) are particularly important since they allow reference to grow from the immediate context toward abstraction by helping infants understand the link between symbols and referents (De Villiers Rader and Zukow-Goldring, 2010). Moreover, deictic gestures seem able to predict linguistic development in both typical and atypical human populations across many cultures (Iverson and Goldin-Meadow, 2005). All these studies suggest that gestures provide a foundation for each new stage in early linguistic development.

A recent discovery in the field of genetics seems providing new insights in support of the gestural-origin theory. In particular, evidence suggests that the *FOXP2* gene, located on the human chromosome 7 (Fisher et al., 1998), could be the molecular substrate linking speech with gesture. In fact, this gene is involved not only in speech production and comprehension but also in gesture coordination.

In an early work Gopnik (1990) argued that the *FOXP2* gene is involved in the development of morphosyntax. For this motivation this gene has been identified more broadly as the “grammar gene” (Pinker, 1994). However, a subsequent investigation suggested that the core deficit associated with the abnormal expression of this gene is one of articulation, with grammatical impairment as secondary outcome (Watkins et al., 2002). Thus, it was proposed (Corballis, 2004) that this gene may play a role in the incorporation of vocal articulation, but have little to do with grammar itself. In support of this suggestion it has been reported that *FOXP2* shows overlapping expression patterns within brains of zebra finches and fetal human brains, particularly in subcortical regions that play important roles in sensorimotor integration and coordinated movements important for vocalization and speech (Teramitsu et al., 2004). Moreover, recent studies on humans extend the role of *FOXP2* gene to the coordination of upper limb movements. For example, the recent study of Peter et al. (2011) found an influence of the *FOXP2* gene on several language processing tasks such as non-word repetition, real word reading efficiency, rapid oral reading. Interestingly, they documented an effect of this gene also on rapid motor sequencing ability which also included finger movements.

Another recent work (Wilcke et al., 2012) has shown that the Single Nucleus Polymorphism (rs12533005) of the *FOXP2* gene can be associated with congenital dyslexia. Interestingly, the difficulty with sequential finger movements is another type of deficit which may characterize reading disorders (Tiffin-Richards et al., 2004). Furthermore, *FOXP2* mutations seem to account for the childhood apraxia of speech (CAS) (MacDermot et al., 2005; Laffin et al., 2012), which is characterized by problems in saying sounds, syllables, and words. Peter (2012) recently described the CAS *FOXP2* phenotype in multi-generational families as characterized not only by deficits in sequential processing at the level of alternating oral motor movements, which is consistent with the traditional CAS definition as a motor programing disorder, but also by deficits in sequential hand movements.

All these studies provide suggestive evidence that the *FOXP2* gene might be the possible molecular substrate linking gestures with verbal language. However, the research in support of this hypothesis is still limited, although there are promising fields of investigation. For example, it would be interesting to assess the impact of the *FOXP2* gene polymorphism on linguistic and manual skills in healthy adults. This investigation not only would provide a further support to the molecular substrate hypothesis for the gestural-origin theory of speech, but it could have also practical implications for developmental and educational psychology, as it might allow an early assessment of the risk for dyslexia and/or dysgraphia in childhood individuals.

Other potential issues worthy of investigation might refer to the study of the expression of *FOXP2* in individuals with special linguistic and/or manual skills (e.g., polyglot people, painters of talent); the influence played by particular socio-environmental factors on its expression, which in turn might influence linguistic and/or manual skills of healthy individuals.

Finally, it would be intriguing to valuate whether the *FOXP2* genetic variations influence the resilience of linguistic and/or manual functions in patients affected by stroke.
